# Beyond the traditional NDVI index as a key factor to mainstream the use of UAV in precision viticulture

**DOI:** 10.1038/s41598-021-81652-3

**Published:** 2021-02-01

**Authors:** Alessandro Matese, Salvatore Filippo Di Gennaro

**Affiliations:** Institute of BioEconomy, National Research Council (CNR-IBE), Via G. Caproni, 8, 50145 Florence, Italy

**Keywords:** Plant sciences, Imaging and sensing, Image processing, Machine learning

## Abstract

In the last decade there has been an exponential growth of research activity on the identification of correlations between vegetational indices elaborated by UAV imagery and productive and vegetative parameters of the vine. However, the acquisition and analysis of spectral data require costs and skills that are often not sufficiently available. In this context, the identification of geometric indices that allow the monitoring of spatial variability with low-cost instruments, without spectral analysis know-how but based on photogrammetry techniques with high-resolution RGB cameras, becomes extremely interesting. The aim of this work was to evaluate the potential of new canopy geometry-based indices for the characterization of vegetative and productive agronomic parameters compared to traditional NDVI based on spectral response of the canopy top. Furthermore, considering grape production as a key parameter directly linked to the economic profit of farmers, this study provides a deeper analysis focused on the development of a rapid yield forecast methodology based on UAV data, evaluating both traditional linear and machine learning regressions. Among the yield assessment models, one of the best results was obtained with the canopy thickness which showed high performance with the Gaussian process regression models (R^2^ = 0.80), while the yield prediction average accuracy of the best ML models reached 85.95%. The final results obtained confirm the feasibility of this research as a global yield model, which provided good performance through an accurate validation step realized in different years and different vineyards.

## Introduction

Characterization of the crop growth response spatial variability plays a key role in achieving site-specific management that optimizes grower inputs on areas much smaller than the entire field. In viticulture the knowledge of “vigour” defined by Winkler et al. (1974)^1^ as the condition expressed in the rapid growth of the parts of the vine, is very useful information for farmers to monitor canopy development and grape production parameters^2^. Since the late 1970s, satellite and aircraft platforms have been widely used in viticulture^3^ for the monitoring of “vigour” spatial variability through the calculation of vegetation indices, based on the canopy spectral response to solar radiation in the visible and near infrared spectrum. However, those platforms offer a limited spatial resolution that does not allow a correct analysis of pure canopy pixels, by removing the inter-row component^4^. In the last decade, the continuous advance in Unmanned Aerial Vehicle (UAV) technologies led to an increasing of spatial ground resolution^5^. Combining powerful photogrammetric software and image analysis methodologies it is possible to perform a geometric analysis at single vine level for canopy height, thickness and volume estimation^6^. As hypothesized by previous works^7,8^, this made it possible to identify new effective solutions to monitor vine vegetative and productive characteristics, with respect to traditional vegetation indices, such as the widely diffused Normalized Difference Vegetation Indices (NDVI). In literature, the use of UAV remote sensing for applications in vineyards is well assessed, however only a few works support remote data with ground truth agronomic measurements made with traditional methods. Those works focus on diseases with ground quantified symptoms^9,10^, water stress with leaf stomatal conductance and leaf water potential^5,11–14^, vegetative development by means of pruning weight and leaf area index (LAI)^4,5,15,16^, leaf compounds as carotenoid content and net photosynthesis^17,18^ and grape quality compounds and yield^5,19–23^. Exploring new approaches for agronomic parameters estimation is a topic of great interest as it would allow to overcome the limitation of traditional time-consuming and destructive measurements, frequently subjective or little representative. With regards to yield prediction, many studies have achieved meaningful results, but the relationships identified have not always been clarified by linear equations. In this context, new Machine Learning (ML) techniques based on non- and semi-parametric structures have become suitable to solve nonlinear and complex problems^24,25^. The most commonly used ML techniques are Support Vector Machine (SVM), Decision Tree (DT), Random Forest (RF), Gaussian Process Regression (GPR) and Artificial Neural Network (ANN). Jeong et al. (2016)^26^ found that RF was highly capable of predicting crop yields in wheat, maize and potato, outperforming multiple linear regression. Romero et al. (2013)^27^ applied several ML methods for the classification of yield components of wheat and showed that the association rule mining method obtained the best performance. Khaki et al. (2020)^28^ described a yield model based on convolutional neural networks (CNNs) and recurrent neural networks (RNNs), which successfully generalized the yield prediction to untested locations for maize (RMSE = 24.10 bushels/acre, validation correlation = 75.04%) and soybean (RMSE = 6.35 bushels/acre, validation correlation = 77.84%) yield. In the field phenotyping area, Herrero‑Huerta et al. (2020)^29^ presented a research focussed on capability of ML techniques to perform grain yield prediction in soybeans by combining data from multispectral and RGB cameras equipped on UAV platforms, achieving an accuracy of over 90.72% by RF and 91.36% by eXtreme Gradient Boosting (XGBoost). Zhou et al. (2020)^30^ compared the model performances for predicting wheat grain yield and protein content between the ML algorithms based on spectral reflectance bands and plant height and the traditional linear regression based on vegetation.

Indices. The research reported that the linear regression model based on the enhanced vegetation index (EVI) provided highest performance capable of predicting the yield with a RMSE = 972 kg/ha, while the RF model based on reflectance bands was capable of predicting the protein content with an RMSE of 1.07%. Unfortunately, there is a lack of this kind of research in viticulture. A recent paper^22^ proposed a methodology based on the combination of spectral (NDVI) and geometric (Fc) UAV indices to establish a relationship with the final yield by using ANN techniques. That research provided interesting results with linear approach models (mean R^2^ = 0.7, mean RMSE = 1.0 k g/vine, mean RE = 23.9%) in the flight closer to harvest in September, but higher predictive accuracy was found with ANN approach (R^2^ = 0.9, RMSE = 0.5 kg/vine, RE = 12.1%). However, low correlation was obtained applying the ANN model to the following season providing a yield overestimation (R^2^ = 0.3, accuracy indicator not reported). That result highlighted the main limitation of the methodology in the seasonal variability, avoiding the opportunity to obtain a global model that returns accurate results for any year. Our work aims to evaluate innovative and accurate forecast methods based on ML techniques applied on high resolution UAV remote sensing data for the prediction of key role agronomic parameters in a sector such as viticulture barely explored with those techniques. Given the significant impact of climate change and terroir on vine physiological response, the added value of the research is represented by the use of a huge dataset considering both the temporal factor by examining 3 very different vegetative seasons, and spatial factor on 3 experimental sites with very different characteristics. Another strength and innovation of the work is the fact of validating the predictive models identified on all the plants present in the study vineyards and not just a few sample plants, thanks to a protocol for the extraction of remote sensing indices as input of the models at the single vine level. The starting point of our research is the performance evaluation of spectral (NDVI) and geometric (canopy thickness and volume) UAV indices with respect to productive (yield and total soluble solids) and vegetative (pruning weight) parameters estimation. The evaluation of these correlations was then deepened by applying an in-depth analysis of the potential of the ML-based models. Finally, considering the importance of yield prediction for farmers^21,31^, the overall goal of this research is the validation of a novel yield forecast method based on a UAV image acquired several weeks before harvest.

## Materials and methods

### Experimental vineyard

**Figure 1:**
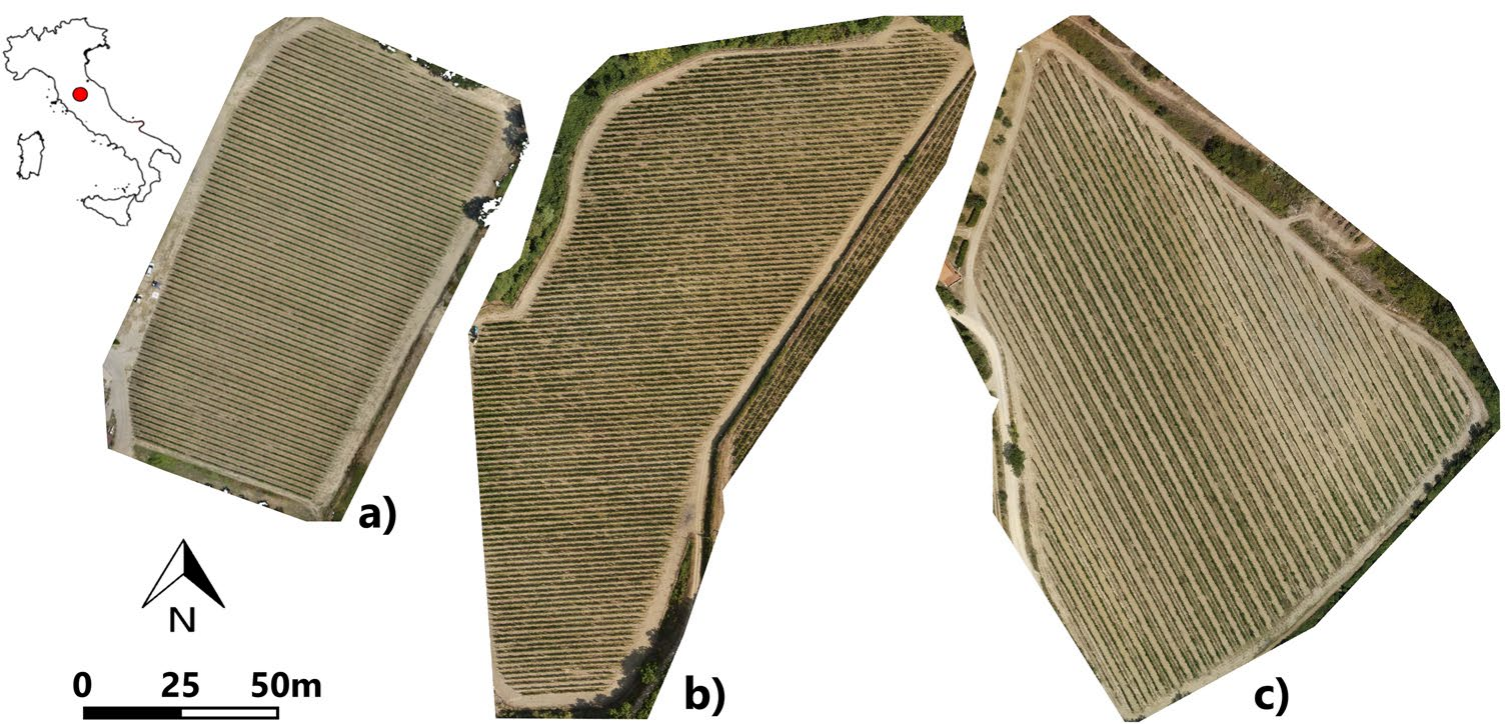
Experimental vineyards: Caggio (**a**), Belvedere (**b**) and Solatio (**c**) sites. The images were created using RGB imagery acquired by UAV during the flight campaign in the 2017 season.

The research was undertaken during the growing seasons 2017, 2018 and 2019 in a 1.4 ha vineyard (355 m above sea level) planted in 2008, owned by Castello di Fonterutoli farm and located near Castellina in Chianti (Siena, Italy) (43° 25′ 45.30″ N, 11° 17′ 17.92″ E) (Fig. 1a). Sangiovese cv. (*Vitis vinifera*) vines were trained with a vertical shoot-positioned trellis system and spur-pruned single cordon. The vine spacing was 2.2 m × 0.75 m (inter-row and intra-row) and the rows were NW–SE oriented on a slight southern slope.

For the validation, two other vineyards were used as independent dataset (only 2019 data), identified as Belvedere (Fig. 1b) and Solatio (Fig. 1c), located in the same area characterized by different conditions with respect to the calibration site. Table 1 summarizes the vineyard features.

**Table 1:** Experimental vineyards.

Site	Caggio	Solatio	Belvedere
Vineyard surface	1.2 ha	2.5 ha	2.2 ha
Row orientation	NW–SE	NW–SE	E–W
Planting year	2008	1999	1997
Grape variety	Sangiovese	Sangiovese	Sangiovese
Rootstock	420A	110R	110R
Vine training system	Cordon spur-pruned	Cordon spur-pruned	Cordon spur-pruned
Vine spacing	2.2 × 0.75	3.0 × 0.9	2.0 × 0.75
Canopy trimming date	11/07/2017	12/07/2019–01/08/2019	02/07/2019
	18/06/2018–23/07/2018		
	24/06/2019		

Vine spacing: distance between rows and between vine along the row. Canopy trimming: management practice which consists of removing the apical part of the shoot containing younger leaves.

### Ground measurements

**Figure 2:**
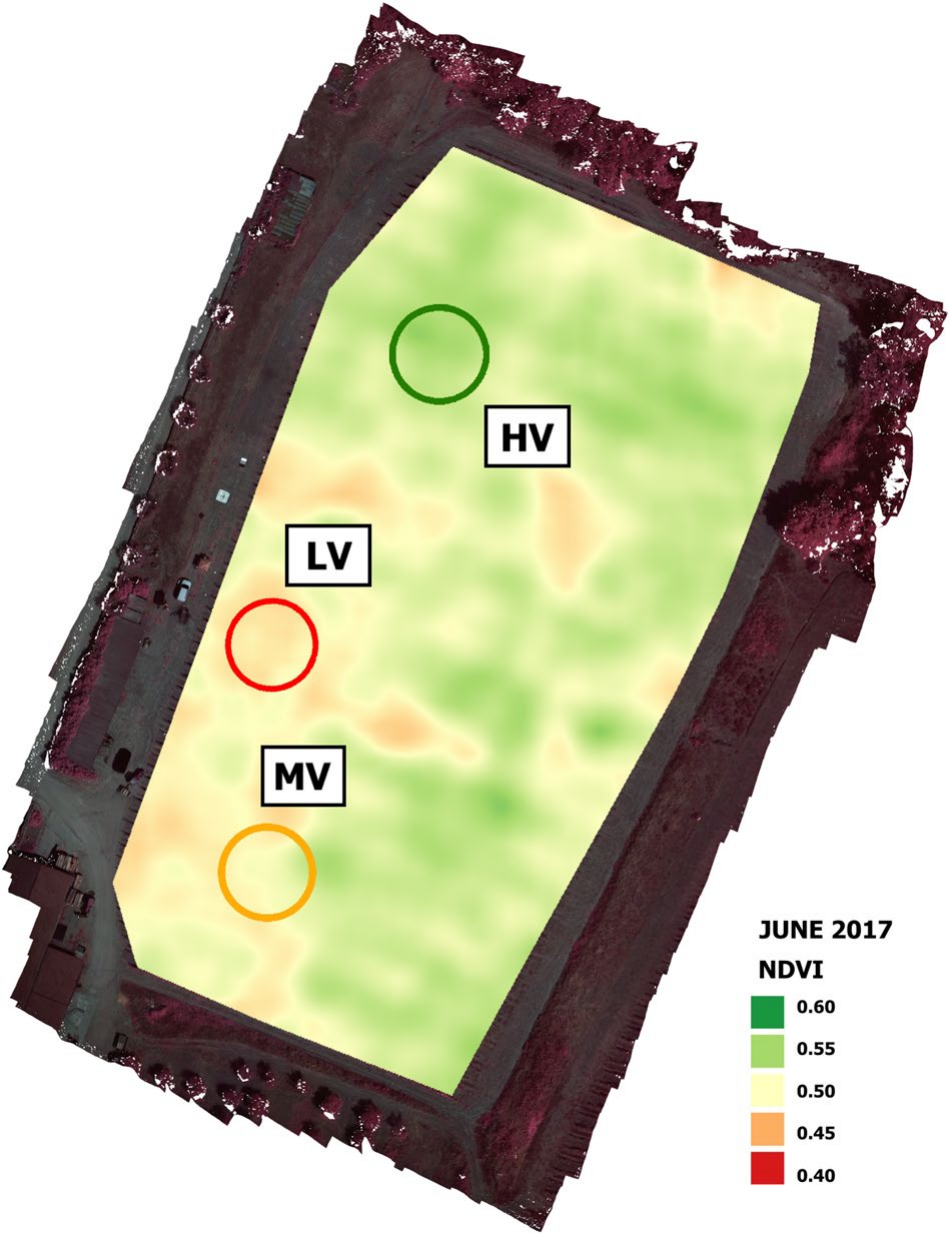
NDVI map created using UAV images acquired during the flight campaign in June 2017 with representative high vigour (HV), medium vigour (MV) and low vigour (LV) zones.

At the beginning of the study, the analysis of multispectral imagery collected in the 2017 season, was used to characterize vigour spatial variability and plan the experimental design (Fig. 2). Representative vine vigour zones were chosen: one with high vigour in the north (HV), a second with low vigour in the middle (LV), and a third with intermediate vegetative behaviour in the south (MV). Within each zone, a sampling area of about 30 × 30 m (0.1 ha) was identified, in which 18 sample plants were identified, for a total of 54 plants within the vineyard. Ground truth measurements related to productive and vegetative parameters were performed every year on each sample vine. At harvest time, yield (kg/vine) and total soluble solids or sugar content (°Brix) were measured for each sampled vine, using a field scale and a hand-held optical refractometer respectively. As indicator of vine vigour, total shoot fresh mass (kg/vine) was determined in the field for each vine in the dormant period following the growing seasons.

### UAV platform and imagery acquisition

Remote sensed images were acquired using a prototype UAV platform consisting of a modified multirotor Mikrokopter (HiSystems GmbH, Moomerland, Germany) (Fig. 1b), described in a previous paper of the authors^21^. The UAV was equipped with an ADC Snap (Tetracam, Inc., Gainesville, FL, USA) multispectral camera, which provides 1.3 MP images in the green (520–600 nm), red (630–690 nm) and NIR (760–900 nm) bands. The use of that camera yielded a ground resolution of 0.03 m/pixel at 50 m above ground level (AGL) flight quote. A series of flight campaigns were performed at the beginning of August of each season in veraison phenological stage (9 August 2017, 8 August 2018 and 6 August 2019).

### Climatic characterization

The climate analysis of each growing season (2017–2018–2019) was conducted by the calculation of some bioclimatic indices, such as sum of daily temperature (min, average and max), number of days with Tmax > 35 °C and cumulative rain in the summer period (June–August), which are based on air temperature and rain parameters collected by a traditional agrometeorological weather station located close to the study vineyard. The elaboration of the bioclimatic indices made it possible to discuss some results relating to the differences in productive and vegetative parameters over the three monitored years.

### Image analysis

Multispectral images acquired by UAV were pre-processed using Agisoft Metashape Professional photogrammetric software (Agisoft LLC, St. Petersburg, Russia), which allows to export the orthomosaic and the digital elevation model (DEM) of the entire vineyard. A vicarious calibration based on the absolute radiance method was chosen, given that the digital number (DN) value for each pixel has a direct relationship (linear model) with the radiance detected by the sensor. For this radiometric calibration process, images from three OptoPolymer (OptoPolymer—Werner Sanftenberg, Munich, Germany) homogeneous and Lambertian surface panels, with 95%, 50% and 5% reflectance, were acquired for each flight.

The filtering procedure of the pure vine canopy pixels was assessed using DEM method with Matlab v.2019a (Mathworks, Natick, MA, USA) as described in Cinat et al. (2019)^32^. The NDVI was computed according to the following Eq. (1):

1 $$NDVI = (NIR - RED)/(NIR + RED)$$

where NIR and RED are the spectral reflectance in near infrared and red bands, respectively.

Geometric variables related to canopy thickness and volume were calculated using the 2.5D methodology with Matlab v.2019a (Mathworks, Natick, MA, USA) described in Di Gennaro and Matese (2020)^6^. Starting from the DEM of vines enclosed within a polygon grid, canopy thickness and vine number are extrapolated through the binarized image of the canopy extracted from each polygon grid. Following that approach, the missing plants present in each vineyard were identified and counted to estimate the yield of the vineyards based on the real vine number (RVN). This operation was fundamental, since a vineyard, once established, commonly loses vines each year due to diseases or abiotic stress. Considering that the number of missing plants can exceed 20%, the decrease in production based on the average production per plant multiplied by the theoretical total number of plants (defined by the vine spacing) can cause a high degree of overestimation.

The NDVI filtered (NDVI_f), canopy thickness (thick) and the canopy volume (Voldem) parameters were extracted for each vine within the vineyard and used as validation dataset. In detail, the real vine number detected by UAV approach respect to original number at planting time were 6264 versus 7273, 7780 versus 9185 and 10,231 versus 14,733 for Caggio, Solatio and Belvedere respectively.

### Yield prediction models based on machine learning approach

Matlab’s Regression Learner app was used to train regression models to predict data yield, total soluble solids and pruning weight parameters. Users can perform automated training to search for the best regression model, including linear regression models, regression trees, Gaussian process regression models, support vector machines, and ensembles of regression trees (Matlab v.2019a). In this work the following regression methods were applied on the full dataset with the aim of taking into account the intra-annual climate variability considering three very different vintages. SVM regression is a nonparametric technique because it relies on kernel functions. Gaussian nonlinear kernel function was also used in this work. DTs are amongst the most intuitively simple classifiers. RF is an ensemble classifier, as it uses many DTs to overcome the weaknesses of a single DT. Boosted DTs are also an ensemble method using DTs. GPR models are nonparametric kernel-based probabilistic models. Exponential kernel function was used in this work. The selected models were applied to identify the performances of the regressions (R^2^, RMSE, MAE, RMSE% and MAE%) with the parameters measured on the ground, considering the filtered NDVI, canopy thickness and volume separately, but also by combining the filtered NDVI factors and canopy thickness, and combining all the factors processed by data acquired by UAV. The RMSE and the MAE indicators were assessed with the following formula:

2 $$RMSE = \sqrt {\frac{1}{n}\sum\nolimits_{j = 1}^n {{{(Predicte{d_j} - Observe{d_j})}^2}} }$$

3 $$MAE = \frac{1}{n}\sum\nolimits_{j = 1}^n {\left| {Predicte{d_j} - Observe{d_j}} \right|}$$

while MAE% and RMSE% were calculated as ratio between MAE and RMSE versus average of observed values.

Having identified the yield as parameter of greatest productive interest, the validation step was assessed only on this parameter by applying the best performing models to estimate the yield (quintals per hectare). The validation was done on Caggio site (Fig. 1a) using a new dataset of remote sensed parameters extracted from all the plants of the vineyard on which the previously developed models (using 54 sampled vines) were applied. Moreover, validation was performed also on two other independent dataset (Belvedere and Solatio vineyard Fig. 1b and 1c). In detail, the validation was made considering the total production of the vineyards measured at harvest. The ML models were tested using a fivefold cross validation. In addition, the model performance indicators were assessed also without cross validation. The models were then applied to a larger dataset, represented by the cumulative production of all plants present in each of the 3 vineyards.

The yield data estimated with the methodology suggested in this paper were compared with the data estimated by the farmer and production data collected during the harvest. The traditional method of yield estimation used by the company was performed a few days before the survey by UAV, and requires visual inspection in representative areas of the vineyard according to the know-how of the agronomist who makes a rapid observation by counting the number of bunches per plant on some vines. Once an average value per plant has been identified, the agronomist multiplies the number of bunches by the average bunch weight, specific to each variety.

## Results and discussion

The results obtained from the research are presented in the following subsections, according to the order defined in the Materials and Methods section.

### Climatic characterization

Table 2 reports several bioclimatic indices, obtained from meteorological data representative of the study site collected by a weather station located on the farm during the three growing seasons (2017–2019).

**Table 2:** Climatic characterization of the 2017–208–2019 seasons by calculation of the sum of daily temperature (min, average and max), number of days with Tmax > 35 °C and cumulative rain in the summer period (June–August).

	2017	2018	2019
Sum of daily min temperature (°C)	1395	1408	1604
Sum of daily average temperature (°C)	2224	2122	2213
Sum of daily max temperature (°C)	3054	2837	2885
Number of days with Tmax > 35 °C (#)	41	12	22
Cumulative rain (mm)	50	152	136

Considering that the summer period is a critical phase for grape production, Table 2 describes very different seasons. In particular, the summer of 2017 was characterized by higher daily maximum temperatures, with 41 days of extreme temperatures above 35 °C, and minimal rainfall (50 mm). In that period, 2018 instead had lower daily maximum temperatures, with the fewest days with extreme temperatures (12 days) and highest rainfall (152 mm). The 2019 season was more temperate, with intermediate values compared to 2017, which was extremely hot and dry, and 2018 cooler and with greater rainfall intensity.

### Ground and remote characterization of vineyard variability

The results of the productive and vegetative characterization performed with destructive ground sampling and UAV images acquisition are summarized in Table 3. The results demonstrated the correct planning of the experimental design, since during the three seasons each vigour zone shows a trend with strong differences in the HV compared to LV zones, and intermediate values in the MV zone.

**Table 3:** Productive and vegetative characterization of vines within each vigour zone during 2017, 2018 and 2019 seasons (mean and standard deviation).

Year	Vigour zone	Yield (kg)	Brix (°)	Pruning weight (kg)	NDVI_filt	vin_thic (m)	Voldem (m^3^)
2017	HV	0.67 ± 0.14	22.53 ± 1.11	0.40 ± 0.07	0.45 ± 0.01	0.33 ± 0.04	0.61 ± 0.10
	MV	0.49 ± 0.14	23.11 ± 0.86	0.24 ± 0.05	0.38 ± 0.05	0.27 ± 0.04	0.39 ± 0.13
	LV	0.32 ± 0.10	25.64 ± 0.83	0.16 ± 0.03	0.34 ± 0.02	0.24 ± 0.03	0.14 ± 0.08
2018	HV	4.12 ± 1.04	18.84 ± 1.61	0.53 ± 0.08	0.56 ± 0.03	0.50 ± 0.03	1.67 ± 0.13
	MV	2.95 ± 0.40	19.87 ± 0.90	0.36 ± 0.03	0.49 ± 0.02	0.42 ± 0.03	1.31 ± 0.12
	LV	1.72 ± 0.28	22.06 ± 0.64	0.32 ± 0.05	0.51 ± 0.02	0.39 ± 0.07	1.15 ± 0.32
2019	HV	3.52 ± 0.72	22.66 ± 1.51	0.48 ± 0.15	0.55 ± 0.01	0.61 ± 0.09	2.01 ± 0.25
	MV	1.86 ± 0.60	23.98 ± 0.69	0.35 ± 0.05	0.48 ± 0.03	0.34 ± 0.07	0.75 ± 0.54
	LV	1.59 ± 0.48	26.16 ± 0.69	0.31 ± 0.04	0.45 ± 0.03	0.31 ± 0.05	0.55 ± 0.23

In the first columns related to ground destructive sampling, higher yield and total fresh biomass values are observed in HV than in LV zones, on the contrary total soluble solids have lower values in HV zone. The ground truth measurements demonstrate the strong climatic impact on vine physiological response. In particular, the hot and dry 2017 season led to a very low yield and minimum vegetative development. While regarding the sugar accumulation, the strong impact of summer abiotic stresses in that season caused a reduction of photosynthetic efficiency and sugar synthesis^23,33^, as a result, lower values than the 2019 season are observed. The 2018 season, with cooler temperatures and higher rainfall, showed higher production and biomass values and lower sugar accumulation in the bunches. The intermediate climatic conditions of 2019 led to yield, quality and vegetative development in line with historical averages.

In the last three columns, Table 3 shows the results of the spectral and geometric elaboration of the UAV data. The images processing outputs confirmed the same trend between the different vigour zones identified by ground-truth measurements. Specifically, the HV zones are characterized by plants with higher values of both NDVI and canopy thickness and volume, while lower values emerge in the LV and intermediate values in the MV areas. In detail, the extreme temperatures and minimum rainfall of 2017 season led to lower values of both NDVI and vegetative growth of the canopy. On the contrary, the peculiarities of 2018 generally translated into greater photosynthetic activity and increased canopy growth. During this season the farm did two canopy trimmings to control the higher shoots growth, but as a consequence lateral growth was stimulated increasing canopy density^34,35^. The rainier and cooler climate of 2018 combined with the double trimming, led to a high vegetative response which significantly reduced heterogeneity within the vineyard. As a consequence, minimal differences between HV and LV zones were observed on all UAV indices. With regards to 2019 season, spectral and geometric indices were in intermediate position between the other years.

### Linear regression between ground measurements and UAV spectral and geometric indices

**Figure 3:**
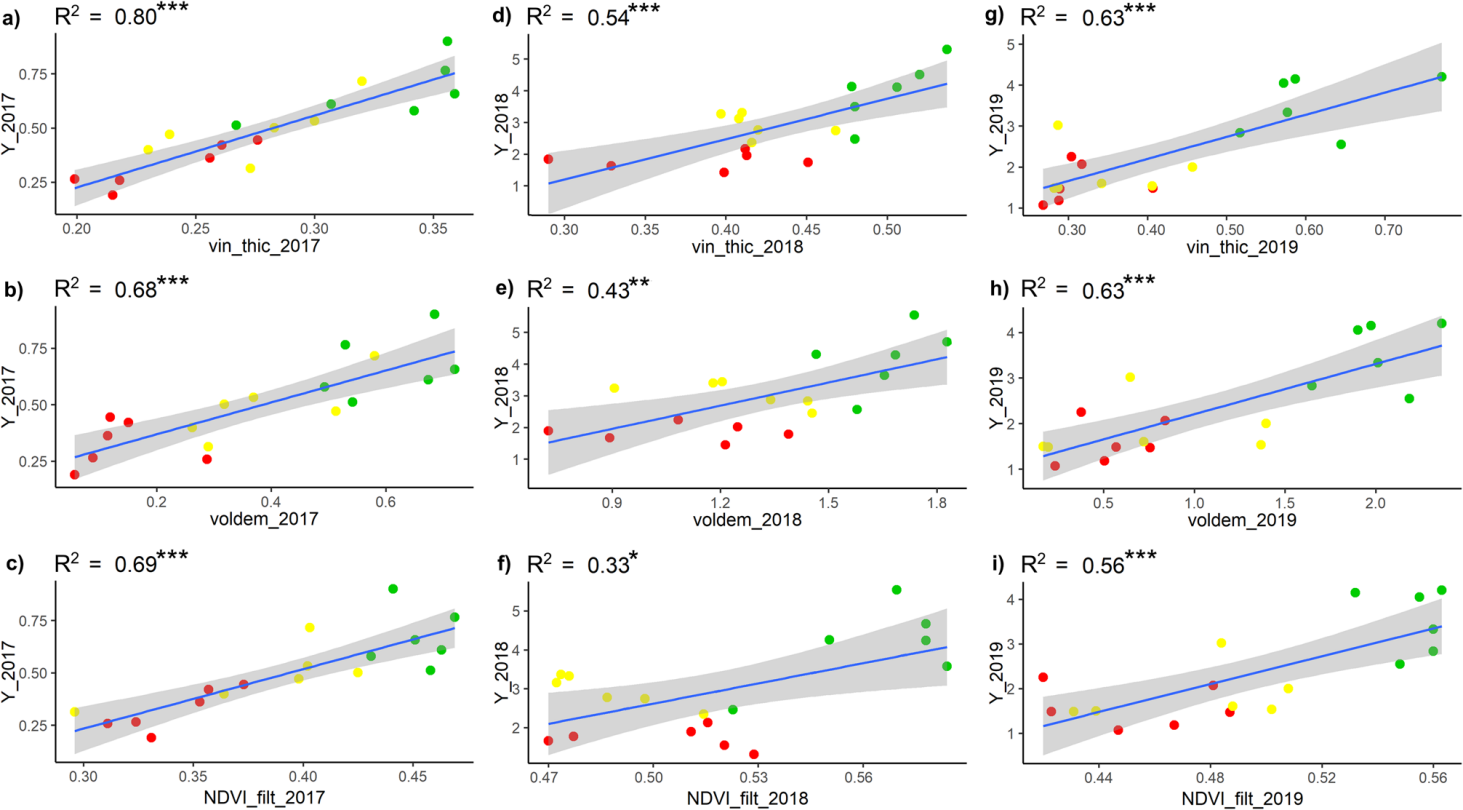
Linear regression results between ground measurements of yield (kg/vine) and UAV remote sensed data, represented by NDVI filtered, canopy thickness (m) and volume (m^3^) in the HV (green dots), MV (yellow dots) and LV (red dots) zones. Significance: ***p < 0.001; **p < 0.01; *p < 0.05.

Figure 3 shows the linear regression results obtained between ground measurements of yield (kg/vine) and UAV spectral and geometric indices. As expected, the yield observed shows positive correlations with all UAV products. In 2017 the best correlation was obtained with the thickness (R^2^ = 0.80), but also the NDVI and volume parameters provide high performance (R^2^ = 0.69 and R^2^ = 0.68 respectively). 2018 presented the lowest correlations for all three parameters, and the thickness is still the most correlated with yield (R^2^ = 0.54). Regarding 2019, all three remotely sensed parameters showed high and significant correlations, higher for thickness and volume (R^2^ = 0.63) with respect to NDVI (R^2^ = 0.56). In general, during the 3 years all the UAV-based parameters presented high significant correlations with yield data (*p* value < 0.001), except for 2018 when NDVI and canopy volume show less significance (*p* value < 0.05). This can be explained as a function of the high vegetative response to climate conditions and canopy management that was observed at the beginning of August, in terms of both photosynthetic efficiency and vegetative development, highlighted by higher NDVI and geometric values respectively.

**Figure 4:**
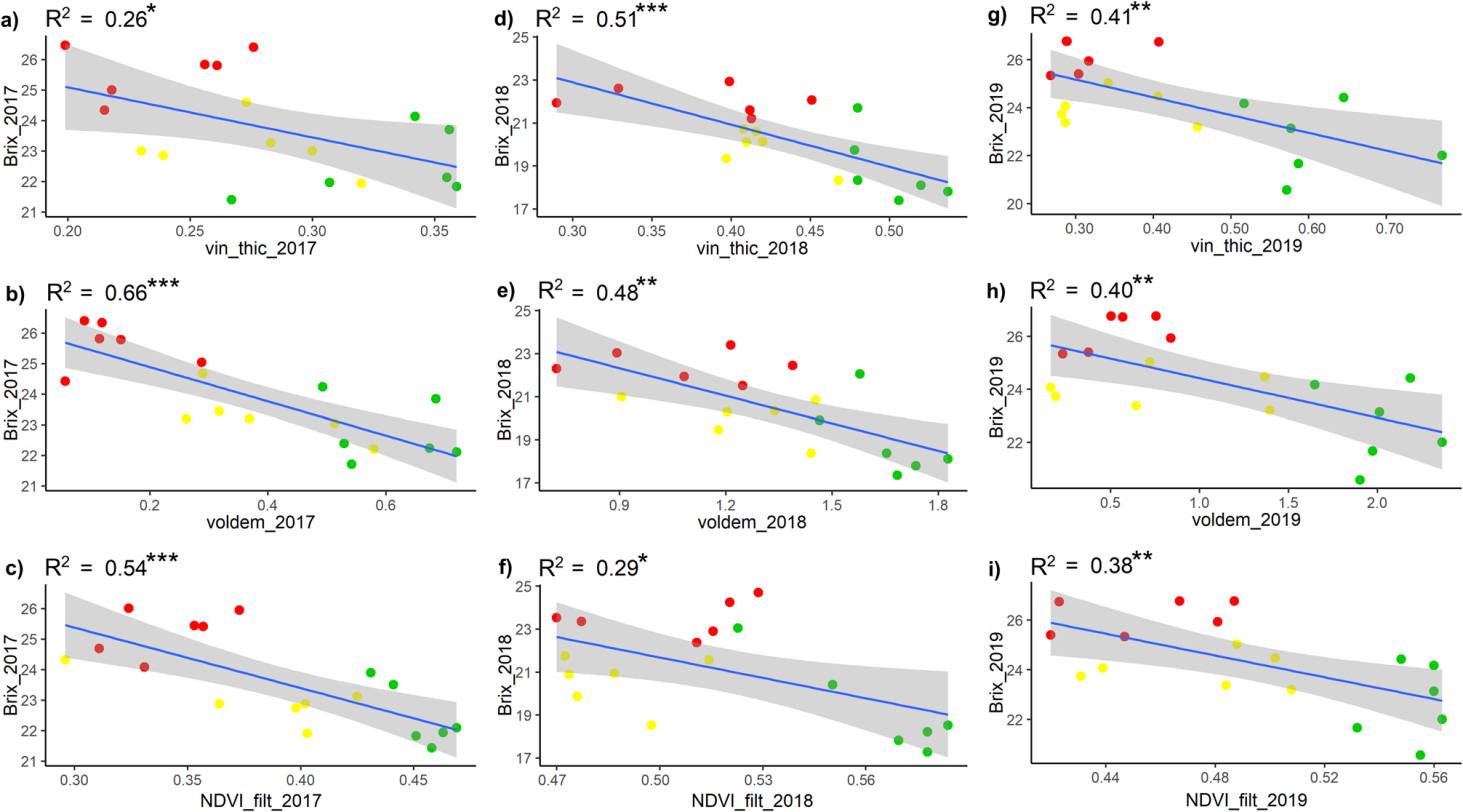
Linear regression results between ground measurements of total soluble solids (°Brix) and UAV remote sensed data, represented by NDVI filtered, canopy thickness (m) and volume (m^3^) in the HV (green dots), MV (yellow dots) and LV (red dots) zones. Significance: ***p < 0.001; **p < 0.01; *p < 0.05.

The linear regressions between sugar content (°Brix) and the UAV indices showed negative correlations, in line with what was expected from the vegetative-productive balance (Fig. 4). As observed for the yield, the geometric indices showed similar or better correlations with respect to the spectral index NDVI. In 2017 the best correlation was obtained with the volume (R^2^ = 0.66), while the thickness showed the lowest coefficient of determination (R^2^ = 0.26). The canopy thickness and volume presented good and similar correlations in 2018 (R^2^ = 0.51 and R^2^ = 0.48 respectively) with respect to the NDVI (R^2^ = 0.29). As for 2019, similar correlations were observed between the three UAV indices (0.38 < R^2^ < 0.41), with lower values for the NDVI. In general, there were lower R^2^ and significance than the regressions obtained with the yield, this may be due to the greater sensitivity of sugar concentration to climatic factors (rainfall, wind, etc.), which may occur even a few days before harvest.

**Figure 5:**
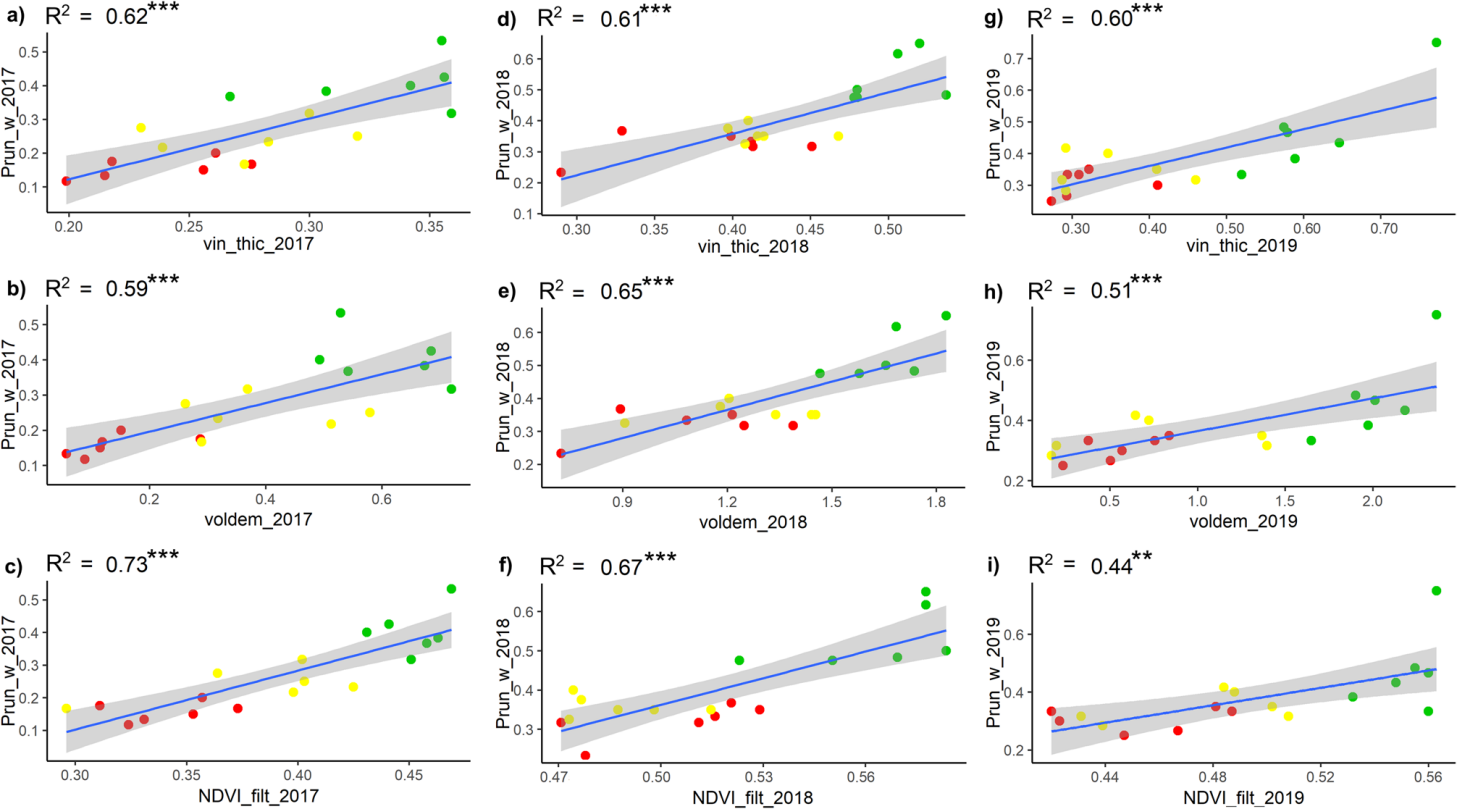
Linear regression results between ground measurements of pruning weight (kg) and UAV remote sensed data, represented by NDVI filtered, canopy thickness (m) and volume (m^3^) in the HV (green dots), MV (yellow dots) and LV (red dots) zones. Significance: ***p < 0.001; **p < 0.01; *p < 0.05.

The results of the linear regressions between pruning weight measurements and UAV data are presented in Fig. 5, which shows positive and significant correlations. In both the 2017 and 2018 seasons, the NDVI presented higher coefficient of determination values (R^2^ = 0.73 and R^2^ = 0.67 respectively) compared to the geometric indices, which showed similar results ranging between R^2^ = 0.59 and R^2^ = 0.65. However, in 2018 the geometric indices provided performances similar to NDVI in biomass estimation. As for the 2019 season, canopy thickness was the most correlated index (R^2^ = 0.60) followed by canopy volume (R^2^ = 0.51), while the NDVI showed lower results (R^2^ = 0.44). Given that the vine considerably slows down vegetative development at the beginning of August with veraison, to concentrate resources on the grape ripening process, the biomass data observed at the end of the season is very representative of the biomass at the time of flight, explaining the excellent correlations with UAV data, with respect to the more dynamic behaviour of the total soluble solids content.

The results between remote spectral and geometric data presented are in line with those reported in the literature. The importance of vegetative growth in terms of vine canopy thickness with respect to spectral information is well assessed by other papers^7,8,36^. Hall et al. (2010)^8^ analysed the relationship between remote descriptors of vine status with the agronomic variables chosen in this work (yield, sugar content and pruning weight). That research identified higher correlation at veraison for the yield with canopy thickness (r = 0.58) reported as CA (canopy area), with respect to spectral data (r = 0.46) referred to as CD (canopy density calculated as mean NDVI values per plant), providing similar but lower correlations than our results. Considering sugar accumulation, that research confirmed the negative correlation with remote indices, although nevertheless lower with respect to this work. Furthermore, Hall et al. (2010)^8^ identified that CA showed higher correlation with pruning weight than CD. In this case, our results identified canopy thickness as a stable descriptor of biomass (0.60 > R^2^ < 0.62), while the NDVI presented different behaviour over the 3 seasons (0.44 > R^2^ < 0.73).

### Machine learning prediction models

Tables 4, 5 and 6 shows the results of application of the ML models on the complete dataset containing the 3 years of data acquired on Caggio vineyard related to ground measurements and UAV indices. The results in terms of coefficient of determination and RMSE, showed the same trend that emerged from the linear regressions applied to the individual years (Figs. 3, 4, 5), namely that the UAV data were more correlated with yield and pruning weight than to the total soluble solids content. In all cases, the linear model provided poorer performance, thus making the selected models interesting to identify a methodology for key parameters estimation in viticulture. Regarding the regressions with the single spectral and geometric UAV data, the linear models showed an average determination coefficient of about 0.66 over the 3 years compared to ML-based models with average values of 0.81. A similar trend was found for pruning weight and total soluble solids with an average R^2^ = 0.59 and 0.32 respectively in the linear models, and R^2^ = 0.71 and 0.48 in the regressive ML models. Taking into consideration the regressions with aggregated values of spectral and geometric UAV parameters, i.e. the NDVI combination with canopy thickness, and the combination of all 3 parameters, Tables 4, 5, 6 always present more performing regressions than those obtained with the individual parameters. The results provided by the application of the different ML models provided similar values of R^2^ and RMSE as regards yield, small differences on the pruning weight, while on the total soluble solids content there were important differences such as the Tree fine model with R^2^ = 0.54 and RF boosted model with R^2^ = 0.32 compared to the NDVI.

**Table 4:** Regression models performance (R^2^, RMSE, MAE, RMSE%, MAE%) obtained from the complete dataset (2017–2018-2019) between yield and NDVI filtered (NDVI_f), canopy thickness (Thick), canopy volume (VolDem), NDVI filtered and canopy thickness (NDVI_f Thick), NDVI filtered, canopy thickness and canopy volume (NDVI_f Thick VolDem). Each cell presents 2 values, the first calculated without cross validation, and the second using fivefold cross validation. The best resulting models are bold typed.

Yield (kg)	NDVI_f	Thick	VolDem	NDVI_f*Thick	NDVI_f*Thick*VolDem
*Linear*					
R^2^	0.65–0.62	0.64–0.63	0.68–**0.64**	0.71–0.66	0.72–0.65
RMSE	0.81–0.84	0.82–0.85	0.77–**0.83**	0.73–0.81	0.72–0.81
MAE	0.68–0.7	0.65–0.68	0.6–**0.65**	0.6–0.65	0.58–0.65
RMSE%	0.42–0.44	0.43–0.44	0.40–0.43	0.38–0.42	0.38–0.42
MAE%	0.36–0.37	0.34–0.36	0.31–0.34	0.31–0.34	0.30–0.34
*Trees fine*					
R^2^	**0.85**–0.7	0.79–0.62	**0.80**–0.53	0.88–0.69	0.88–0.57
RMSE	**0.53**–0.74	0.62–0.86	**0.61**–0.94	0.48–0.77	0.47–0.9
MAE	**0.39**–0.58	0.48–0.69	**0.43**–0.76	0.34–0.58	0.34–0.68
RMSE%	0.28–0.39	0.32–0.45	0.32–0.49	0.25–0.4	0.25–0.47
MAE%	0.20–0.30	0.25–0.36	0.22–0.4	0.18–0.3	0.18–0.36
*RF boosted*					
R^2^	0.84–**0.73**	0.79–**0.67**	0.80–0.63	0.87–**0.73**	0.88–0.71
RMSE	0.54–**0.71**	0.62–**0.8**	0.61–0.84	0.48–**0.72**	0.47–0.75
MAE	0.42–**0.57**	0.48–**0.65**	0.47–0.69	0.35–**0.57**	0.33–0.59
RMSE%	0.28–0.37	0.32–0.42	0.32–0.44	0.25–0.38	0.25–0.39
MAE%	0.22–0.3	0.25–0.34	0.25–0.36	0.18–0.3	0.17–0.31
*SVM fine Gaussian*					
R^2^	0.80–0.6	0.79–0.51	0.80–0.55	**0.91**–0.52	**0.91**–0.6
RMSE	0.60–0.87	0.62–0.97	0.61–0.92	**0.41**–0.96	**0.41**–0.87
MAE	0.45–0.67	0.46–0.74	0.44–0.73	**0.29**–0.81	**0.28**–0.7
RMSE%	0.31–0.45	0.32–0.51	0.32–0.48	0.21–0.5	0.21–0.45
MAE%	0.23–0.35	0.24–0.39	0.23–0.38	0.15–0.42	0.15–0.37
*Gaussian exp GPR*					
R^2^	0.83–0.7	**0.80**–0.63	0.80–0.59	0.89–0.72	0.9–**0.71**
RMSE	0.55–0.75	**0.60**–0.84	0.6–0.88	0.44–0.73	0.42–**0.74**
MAE	0.43–0.58	**0.49**–0.69	0.49–0.72	0.33–0.57	0.32–**0.56**
RMSE%	0.29–0.39	0.31–0.44	0.31–0.46	0.23–0.38	0.22–0.39
MAE%	0.22–0.3	0.26–0.36	0.26–0.38	0.17–0.3	0.17–0.29

**Table 5:** Regression model’s performance (R^2^, RMSE, MAE, RMSE%, MAE%) obtained from the complete dataset (2017–2018–2019) between pruning weight and NDVI filtered (NDVI_f), canopy thickness (Thick), canopy volume (VolDem), NDVI filtered and canopy thickness (NDVI_f Thick), NDVI filtered, canopy thickness and canopy volume (NDVI_f Thick VolDem). Each cell presents 2 values, the first calculated without cross validation, and the second using fivefold cross validation. The best resulting models are bold typed.

Pruning weight (kg)	NDVI_f	Thick	VolDem	NDVI_f*Thick	NDVI_f*Thick*VolDem
*Linear*					
R^2^	0.63–0.61	0.59 -0.57	0.55–0.52	0.68–0.61	0.69–0.66
RMSE	0.07–0.08	0.08–0.08	0.08–0.09	0.07–0.08	0.07–0.07
MAE	0.06–0.06	0.07–0.06	0.07–0.07	0.05–0.06	0.06–0.06
RMSE%	0.20–0.23	0.23–0.23	0.23–0.26	0.20–0.23	0.20–0.20
MAE%	0.17–0.17	0.20–0.17	0.20–0.20	0.14–0.17	0.17–0.17
*Trees fine*					
R^2^	**0.80–**0.58	0.71–0.55	0.72–0.4	**0.85–**0.58	0.85–0.52
RMSE	**0.06–**0.08	0.07–0.08	0.07–0.1	**0.05–**0.08	0.04–0.09
MAE	**0.04**–0.06	0.05–0.06	0.05–0.08	**0.03–**0.06	0.03–0.07
RMSE%	0.17–0.23	0.20–0.23	0.20–0.29	0.14–0.23	0.11–0.26
MAE%	0.11–0.17	0.14–0.17	0.14–0.23	0.09–0.17	0.09–0.20
*RF boosted*					
R^2^	0.70–0.56	0.67–**0.58**	0.68–0.51	0.75–**0.64**	0.78–**0.67**
RMSE	0.07–0.08	0.07–**0.08**	0.07–0.09	0.06–**0.07**	0.06–**0.07**
MAE	0.05–0.06	0.05–**0.05**	0.05–0.07	0.04–**0.05**	0.04–**0.05**
RMSE%	0.20–0.23	0.20–0.23	0.20–0.26	0.17–0.20	0.17–0.20
MAE%	0.14–0.17	0.14–0.14	0.14–0.20	0.11–0.14	0.11–0.14
*SVM fine Gaussian*					
R^2^	0.69–0.52	0.69–0.53	0.63–**0.54**	0.72–0.44	0.75–0.35
RMSE	0.07–0.08	0.07–0.08	0.07–**0.08**	0.06–0.09	0.06–0.1
MAE	0.05–0.06	0.05–0.07	0.05–**0.06**	0.04–0.07	0.03–0.07
RMSE%	0.20–0.23	0.20–0.23	0.20–0.23	0.17–0.26	0.17–0.29
MAE%	0.14–0.17	0.14–0.20	0.14–0.17	0.11–0.20	0.09–0.20
*Gaussian exp GPR*					
R^2^	0.74–**0.63**	**0.79–**0.41	**0.75–**0.45	0.84–0.59	**0.86–**0.58
RMSE	0.06–**0.07**	**0.06–**0.09	**0.06–**0.09	0.05–0.08	**0.04–**0.08
MAE	0.05–**0.05**	**0.04–**0.06	**0.05–**0.07	0.04–0.05	**0.03–**0.06
RMSE%	0.17–0.20	0.17–0.26	0.17–0.26	0.14–0.23	0.11–0.23
MAE%	0.14–0.14	0.11–0.17	0.14–0.20	0.11–0.14	0.09–0.17

**Table 6:** Regression model’s performance (R^2^, RMSE, MAE, RMSE%, MAE%) obtained from the complete dataset (2017–2018–2019) between total soluble solids and NDVI filtered (NDVI_f), canopy thickness (Thick), canopy volume (VolDem), NDVI filtered and canopy thickness (NDVI_f Thick), NDVI filtered, canopy thickness and canopy volume (NDVI_f Thick VolDem). Each cell presents 2 values, the first calculated without cross validation, and the second using fivefold cross validation. The best resulting models are bold typed.

Brix (°)	NDVI_f	Thick	VolDem	NDVI_f*Thick	NDVI_f*Thick*VolDem
*Linear*					
R^2^	0.33–**0.28**	0.26–0.22	0.36–0.36	0.34–0.28	0.40–0.36
RMSE	1.97–**2.13**	2.09–2.18	1.96–2.01	1.99–2.12	1.90–2.01
MAE	1.66–**1.79**	1.75–1.82	1.65–1.67	1.66–1.75	1.55–1.65
RMSE%	0.09–0.09	0.09–0.1	0.09–0.08	0.09–0.09	0.08–0.09
MAE%	0.07–0.08	0.08–0.08	0.07–0.07	0.07–0.08	0.07–0.07
*Trees fine*					
R^2^	**0.54–**0.09	**0.56–**0.24	**0.69–**0.38	0.63–0.30	0.65–0.20
RMSE	**1.67–**2.39	**1.62–**2.15	**1.35–**1.96	1.50–2.08	1.45–2.24
MAE	**1.29–**2.01	**1.34–**1.79	**1.07–**1.64	1.22–1.78	1.13–1.88
RMSE%	0.07–0.11	0.07–0.09	0.06–0.09	0.07–0.09	0.06–0.1
MAE%	0.06–0.09	0.06–0.08	0.05–0.07	0.05–0.08	0.05–0.08
*RF boosted*					
R^2^	0.32–0.14	0.37–0.32	0.38–0.34	0.46–0.30	0.50–0.34
RMSE	2.01–2.31	1.93–2.03	1.92–2.03	1.79–2.08	1.72–2.04
MAE	1.54–1.93	1.47–1.71	1.44–1.72	1.42–1.73	1.35–1.71
RMSE%	0.09–0.1	0.08–0.09	0.08–0.09	0.08–0.09	0.08–0.09
MAE%	0.07–0.08	0.06–0.08	0.06–0.08	0.06–0.08	0.06–0.08
*SVM fine Gaussian*					
R^2^	0.40–0.22	0.46–0.19	0.60–0.30	**0.66–0.32**	**0.69–**0.32
RMSE	1.89–2.21	1.79–2.22	1.55–2.09	**1.42–2.07**	**1.35–**2.06
MAE	1.42–1.9	1.26–1.76	1.18–1.76	**0.88–1.70**	**0.78–**1.65
RMSE%	0.08–0.1	0.08–0.1	0.07–0.09	0.06–0.09	0.06–0.09
MAE%	0.07–1.9	0.06–0.08	0.06–0.08	0.06–0.07	0.06–0.07
*Gaussian exp GPR*					
R^2^	0.37–0.20	0.47–**0.35**	0.55–**0.40**	0.57–0.31	0.57–**0.40**
RMSE	1.95–2.24	1.78–**1.99**	1.63–**1.93**	1.59–2.07	1.59–**1.94**
MAE	1.58–1.86	1.51–**1.69**	1.36–**1.61**	1.32–1.71	1.33–**1.66**
RMSE%	0.09–0.1	0.08–0.09	0.07–0.08	0.07–0.09	0.07–0.09
MAE%	0.07–0.08	0.07–0.07	0.06–0.08	0.06–0.08	0.06–0.07

Considering the yield parameter as the most relevant for farmers, an in-depth analysis of the potential of the ML models for yield forecast was conducted. The most commonly used linear model was compared with the most performing ML model identified in Table 4. Furthermore, given the similar performances of canopy thickness and volume, it was decided to use the first parameter as it required lower computing power and processing time to be elaborated from UAV images. Table 7 therefore shows the results of the selected models with and without cross validation for the yield forecast on Caggio (2017–2018–2019) and Solatio and Belvedere (2019) vineyards.

**Table 7:** Yield models’ output (quintals per hectare) related to real plant number without missing plants (Yield RVN) using NDVI filtered (NDVI_f), canopy thickness (Thick), NDVI filtered and canopy thickness (NDVI_f Thick) compared to yield ground estimation and yield harvested. The best resulting models are bold typed.

	Year	Vineyard	NDVI_f		Thick		NDVI_f Thick		Yield ground estimation	Yield harvested
			Linear	Trees	Linear	Exp GPR	Linear	SVM		
Yield RVN (best models without cross validation)	2017	Caggio	37.3	35.8	46.5	**42.9**	35.7	48.5	49.1	44.3
	2018	Caggio	136.8	132.1	115.5	116.8	129.1	**114.3**	73.6	71.1
	2019	Caggio	126.2	116.4	118.0	**114.0**	124.5	106.6	78.5	110.8
	2019	Solatio	100.0	112.7	65.4	**64.7**	85.5	70.8	70.0	53.6
	2019	Belvedere	145.2	161.8	83.3	82.2	117.6	97.2	69.3	73.7
			RF boosted		RF boosted		RF boosted			
Yield RVN (best model with fivefold cross calibration)	2017	Caggio	34.1		40.1		36		49.1	44.3
	2018	Caggio	134		116		128.2		73.6	71.1
	2019	Caggio	118.6		115		118.2		78.5	110.8
	2019	Solatio	109.3		64.8		90.5		70.0	53.6
	2019	Belvedere	157.5		**81.3**		126.1		69.3	73.7

The yield is strongly affected by the number of missing plants present in the vineyard for reasons related to senescence, mechanical damage or wood diseases, such as the widespread esca disease^9^, however it is not easy for the farmer to have the knowledge of that number, since a visual count walking along all the rows every year would be extremely time-consuming. Considering the accurate missing vine detection by our approach, the RVN yield data estimated by UAV imagery are considerably more accurate than the values estimated by the agronomist by means visual ground observation, confirming the good performance of the post processing workflow in the recognition and counting of missing plants along the rows.

The yield estimation by visual observations in the vineyard responded with good accuracy to the real production harvested. However, in 2019 season the ground estimation provided important errors on the three vineyards examined, probably due to low representative vines chosen for ground observation. As expected by previous results, both spectral and geometric indices provided similar yield estimation values, however it is observed that models based on a single NDVI input variable showed lower accuracy, than single use of the canopy thickness or combined with NDVI. Furthermore, models based on single NDVI didn’t present a clear behaviour, while the linear model demonstrated lower predictive performance than the exp GPR and SVM models, applied on single canopy thickness and combined with NDVI respectively. Considering the performance of these models, the single use of canopy thickness input variable led to higher accuracy than combined with NDVI, with the exception of the 2018 season. This could be a consequence of the greater stability of the geometrical indices, compared to the high sensitivity of the spectral data that is affected by the light environmental conditions and the heterogeneous structure of the top of the canopy, due to leaves with different angle and orientation and mixed light and shadow conditions within the same canopy. However, it was an anomalous year with two canopy trimmings, the second approximately 17 days before the UAV flight, which strongly stimulated the vine vegetative response resulting in a huge development of secondary shoots. The high values of both NDVI for the spectral response of the youngest leaves and the increase in canopy volume caused a yield overestimation in all the models. The experience gained in this work has highlighted the importance of monitoring time, in particular in a year with vegetative excesses such as 2018, the survey should have been done a few days after canopy trimming to avoid the impact of the strong vegetative response. The exp GPR model with canopy thickness values as input data was the best performing, with an average error over the 3 years in Caggio and for 2019 in Solatio and Belvedere of about 20.5% compared to the estimates made by visual observations on the ground that provided a 16.0% error. However, excluding the error obtained from the methodology suggested in the year 2018 (64.3%), the accuracy of the yield forecast methodology by UAV platform significantly increases, presenting an error of 9.6%, therefore a better estimate than the traditional method.

The most accurate models for yield forecast in Belvedere site was RF boosted selected after a k-fold cross validation, while the best accuracy in the other vineyards (Caggio and Solatio) was reached using models selected without cross validation. Indeed, the best overall score might not be the best model for yield forecast and sometimes a model with slightly lower overall score could be the better model.

In the literature there is only a similar work proposed by Ballesteros et al. (2020)^22^, which described a multitemporal analysis using spectral and geometric data elaborated from multispectral UAV sensing, for the development of a yield predictive model based on linear and ANN regressions. The linear regressions for the 2017 season showed that better correlations were identified in September close to the harvest, and the best model suggested is the one with multiple linear regression for all flight dates with final yield reaching R^2^ = 0.76 with the pure canopy pixel NDVI (NDVI_WIV_), then similar results with fraction vegetation cover (Fc) or both combined (NDVI_WIV x Fc_) (R^2^ = 0.71). That work therefore presented a different trend to what emerged from our research in which the filtered NDVI data (NDVI_f) and canopy thickness (Thick) provided a linear regression with similar coefficient of determination as single variables (R^2^ = 0.65 and R^2^ = 0.64 respectively), while better results when combined together (R^2^ = 0.71). Taking the RMSE data into consideration, the most accurate estimated yield values were provided by the NDVI_f xThick model identified in our work (RMSE = 0.73 kg/vine) compared to the NDVI_WIV_ model of the cited work (RMSE = 0.81 kg/vine). With regard to the results obtained with the application of predictive ANN models applied on the combined spectral and geometric information factors, both researches showed better performing outputs in terms of both R^2^ and RMSE compared to traditional regressions. In this respect, the data obtained from the cited research, was slightly better than the results presented in this paper. This aspect is probably due to the fact that the analysis done by Ballesteros et al. (2020)^22^ was related to a single year and wasn’t affected by inter-annual variability, while our research takes into consideration three different seasons, which however make the model much more robust as subsequently demonstrated by the validation results on two other vineyards at the end of the 3 years of experimentation. The validation of the models analysed is much more structured than that described in the paper of Ballesteros et al. (2020)^22^, where a survey made the following year on the same experimental site was used, while there were 3 different study sites in this work. The models calibrated over 3 years were efficient on all vineyards with estimated production values close to those observed, while the research by Ballesteros et al. (2020)^22^ by applying the model calibrated in 2017 on the same study site the following year, provided a lower coefficient of determination (R^2^ = 0.32) and an overestimation with almost double yield values per vine. The results obtained from 3 years of experimentation on such different years, have allowed to overcome the limits in the cited work on the difficulty of identifying global predictive production models using only 1 year of calibration due to inter-annual yield and canopy variability.

### Overall evaluation

The NDVI index becomes an excellent indicator of vegetative vigour taking into consideration intensive crops with horizontal development, such as cereals or horticulture, providing combined information on vegetative activity (photosynthesis) and growth density (biomass). The limit of this index emerges when the vigour of discontinuous tree crops, such as vines, olives and pome fruits has to be assessed. In this condition, the soil component with respect to canopy cover is relevant and above all the vertical development is key factor for vegetative growth assessment. In these crops, the leaf spectral response of the canopy top describes only a part of the vigour of the plant, as all the vegetative growth component is lost (canopy width, height and volume). Furthermore, there are a large number of factors that can affect the quality of spectral data: stable light conditions, sun radiation angle, radiometric correction know-how, leaf status such as age, mechanical damage and symptoms due to biotic and abiotic stresses.

Today, the high spatial resolution obtained thanks to the use of UAV opens new possibilities in the study and characterization of vigour, allowing filtering techniques to be applied and pure canopy pixel spectral data extracted. At the same time, the application of SfM algorithms on a UAV dataset with high overlap level led to a volumetric reconstruction of the canopy directly linked to vegetative growth and biomass. Following this approach, it becomes possible to use high resolution multispectral images to individually quantify the components of the photosynthetically active biomass (PAB), obtaining the spectral response of the canopy from 2D analysis and biomass data from 3D analysis, thus overcoming the limit of the traditional NDVI index in the characterization of vigour. In support of the geometric approach is added the fact that it can be obtained with common RGB cameras, with minimum costs and very high performance derived from the extreme spatial resolution^6^. In general, the geometric approach presented in this paper can be applied on many tree crops, however on some exceptions with full soil covering such as overhead trellis system^21^ and on extended crops such as horticultural crops or cereals, the NDVI index remains the only applicable solution for estimating production and vegetative parameters.

## Conclusions

In the last decade there has been an exponential growth in research activity on the identification of correlations between vegetational indices elaborated by a multispectral camera mounted on UAVs and productive and vegetative parameters of the vine. Our results underlined the great potential of ML approaches to predict several weeks before harvest the yield, which is the agronomic parameter that mainly drive the economic return for the farmer. Compared to previous works, our results were supported by a robust dataset that examined both climatic variability working on 3 different years and different field conditions using 3 study sites. The model based on geometric data presents better results than the use of NDVI data; this opens up an extremely interesting perspective to the proposed methodology by focusing on RGB sensors compared to multispectral cameras. Among the main advantages, production estimates could be made using very simple and inexpensive instrumentation (< 1000.00 €), overcoming any problems given the need for spectral know-how on radiometric correction and data analysis, primarily for filtering the canopy with low-temperature sensors resolution from common multispectral cameras (< 3MP), but also in data interpretation due to the complex structure of the canopy as a consequence of the copresence of leaves fully exposed to the sun, partially or completely shaded, turned leaves and gaps that reveal the soil below. A limitation of the work is the identification of sample plants used to evaluate the performance of the methodology in the estimation of productive and vegetative parameters. In particular, the plants were not chosen spatially distributed within the vineyard but concentrated in areas representative of spatial variability. Consequently, it was not possible to use a geostatic analysis approach, however this study aims to evaluate traditional statistical methods of comparison commonly used in agronomic experiments.
